# Ketone Body *β*-Hydroxybutyrate Prevents Myocardial Oxidative Stress in Septic Cardiomyopathy

**DOI:** 10.1155/2022/2513837

**Published:** 2022-03-18

**Authors:** Liwei Ji, Qinqin He, Yinghai Liu, Yan Deng, Maodi Xie, Kaiteng Luo, Xintian Cai, Yunxia Zuo, Wei Wu, Qian Li, Ronghua Zhou, Tao Li

**Affiliations:** ^1^Department of Anesthesiology, Laboratory of Mitochondria and Metabolism, National Clinical Research Center for Geriatrics, West China Hospital of Sichuan University, Chengdu 610041, China; ^2^Department of Anesthesiology, The General Hospital of Western Theater Command, Chengdu 610083, China

## Abstract

Septic cardiomyopathy is a life-threatening complication of severe sepsis and septic shock. Oxidative stress and mitochondrial dysfunction have been identified as significant abnormalities in septic cardiomyopathy. However, specific treatments are rare. This study aims to investigate the impact of *β*-hydroxybutyrate (*β*-OHB) on septic cardiomyopathy and explore the underlying mechanism(s). We found that pretreatment of D-*β*-hydroxybutyrate-(R)-1,3 butanediol monoester (ketone ester, 3 mg/g body weight, once daily) by gavage for three days elevated the levels of ketone bodies, especially that of *β*-hydroxybutyrate (*β*-OHB) in the circulation and mouse hearts, which exerted a protective effect against lipopolysaccharide (LPS, 20 mg/kg)-induced septic cardiomyopathy in mice. In addition, an LPS-stimulated macrophage-conditioned medium (MCM) was used to mimic the pathological process of septic cardiomyopathy. Mechanistically, *β*-OHB alleviated myocardial oxidative stress and improved mitochondrial respiratory function through the antioxidant FoxO3a/MT2 pathway activated via histone deacetylase (HDAC) inhibition, which ultimately enhanced heart performance in septic cardiomyopathy. Our results, therefore, suggested an unappreciated critical role of *β*-OHB in septic heart protection as well as highlighted the potential of *β*-OHB as a simple remedy for the septic cardiomyopathy population.

## 1. Introduction

Sepsis is the leading cause of death among intensive care unit patients and is estimated to affect over 30 million people annually worldwide [1, 2]. Cardiac dysfunction caused by sepsis, which is defined as sepsis-induced cardiomyopathy, is an acute complication of severe sepsis and increases the mortality risk substantially [3, 4]. Septic cardiomyopathy is characterized by impaired left ventricular systolic and diastolic functions [5–7]. However, the pathogenesis of septic cardiomyopathy remains obscure [3, 8]. The treatment for this fatal condition remains largely supportive, and the therapeutic implications are uncertain [4, 9]. Hence, to manage the condition, it is imperative to explain the pathophysiological processes of septic cardiomyopathy and devise efficacious approaches.

Mitochondrial dysfunction and oxidative stress are increasingly recognized as the critical factors of septic myocardial injury [2, 8, 10]. The heart is an organ with high energy requirements and depends chiefly on mitochondrial oxidative phosphorylation (OXPHOS) [11, 12]. Mitochondria, the energy centers of cardiomyocytes, control cardiomyocyte metabolism and survival by constantly supplying ATP [13, 14]. Impaired mitochondrial respiratory function obstructs the energy supply and thus fails to support heart function in septic cardiomyopathy [7, 9, 15]. More importantly, mitochondria are a rich source of reactive oxygen species (ROS) in mammalian cells [14]. Under oxidative stress, increased ROS levels can damage mitochondrial membranes protein and DNA, thereby impairing the ability of the mitochondria to generate ATP [14, 16]. However, it is challenging to link these abnormalities to therapeutic strategies and relevant clinical outcomes. Therefore, antioxidant therapy targeting the mitochondrial function may facilitate the treatment of septic cardiomyopathy.

Several evidences highlight the crucial roles of ketone bodies in the pathophysiological progression of cardiovascular diseases, including heart failure, myocardial fibrosis, and vascular endothelial injury [17–20]. Ketone bodies include *β*-hydroxybutyrate (*β*-OHB), acetoacetate, and acetone [21]. In certain physiological states, such as fasting, starvation, and low-carbohydrate diets, ketone bodies become a significant source of overall energy and the substrate of mammalian metabolism in the extrahepatic tissues [21, 22]. In addition to acting as an alternative energy source, the ketone body *β*-OHB can serve as an endogenous histone deacetylase (HDAC) inhibitor, which is associated with increased global histone acetylation to initiate the transcription of antioxidant genes in response to oxidative stress [23]. Ketone ester (KE) could cause a rapid and significant increase in the blood D-*β*-OHB levels, and its production and commercialized has increased over the past decade. In light of the regulatory effects of *β*-OHB on oxidative stress, it is worthwhile to explore its cardioprotective effect and therapeutic potential in fatal septic cardiomyopathy based on redox homeostasis and mitochondrial performance.

In this study, we attempted to investigate the impacts of *β*-OHB on septic cardiomyopathy and elucidate the underlying mechanisms. By using animal models and cardiomyocytes to simulate the process of septic cardiomyopathy, the role of *β*-OHB as a HDAC inhibitor was demonstrated. *β*-OHB activated the antioxidant pathways to alleviate myocardial oxidative stress and improved the mitochondrial respiratory function, which ultimately enhanced the cardiac performance in septic cardiomyopathy.

## 2. Materials and Methods

### 2.1. Animal Care

All animal procedures were conducted in compliance with protocols approved by the Institutional Animal Care and Use Committee (IACUC) of the West China Hospital, Sichuan University, China (Protocol #20211406A). All animals were cared for in accordance with the recommendations provided in the *Guide for the Care and Use of Laboratory Animals 8^th^ Edition*. Healthy male C57BL/6 J mice aged 8 to 10 weeks were purchased from GemPharmatech Experimental Animal Centre, China. The mice were kept under specific pathogen-free (SPF) conditions at a constant temperature of 22°C on a 12 : 12 h light/dark cycle with free access to food and water. The septic mouse model was established as described previously [24, 25]. As female C57BL/6 J mice were reportedly resistant to cardiac injury in sepsis, we challenged male mice with a high dose of *Escherichia coli* lipopolysaccharide (LPS, 20 mg/kg, i.p., Sigma) to prepare septic cardiomyopathy models [5, 26]. The KE (D-*β*-hydroxybutyrate-R 1,3-butanediol monoester, KE) in this study is provided by a commercial drink from the T△S® Global company (Thame, UK) that contains 25 g KE in 59-mL purified water (pH~3.0) [27, 28]. Male C57BL/6 J mice aged 8–10 weeks were randomly categorized into three groups, namely, the control group (Control), LPS group (LPS), and LPS + KE pretreatment group. In the LPS + KE group, the mice received KE (3 mg/g body weight/day for three days) via oral gavage. In the control and LPS groups, the mice received an equivalent amount of saline for three days. Next, the LPS and LPS + KE groups mice were treated with LPS 30 min after administering the last dose of KE [28, 29]. After 6 h of LPS injection, the cardiac functions were assessed by 2-dimensional (2D) echocardiography. The hearts and blood collected from the mice in each group were used for further analyses. No death was recorded in this study within 6 h after the administration of LPS. No animals were intentionally excluded from the analyses.

### 2.2. Cell Culture

RAW264.7 murine macrophage cells were obtained from the American Type Culture Collection (ATCC; Rockville, MD, USA). The cells were cultured in Dulbecco's Modified Eagle's medium (DMEM; HyClone) (High Glucose) containing 1 g/L glucose, 4 mM glutamine, 110 mg/L sodium pyruvate supplemented with 10% (*v*/*v*) fetal bovine serum (FBS; Biological Industries), 100 U/mL penicillin, and 100 mg/mL streptomycin (both from HyClone Laboratories Inc.) under a humidified atmosphere of 5% CO_2_ at 37°C. Referring to our previous protocol, RAW264.7 macrophage cells were treated with LPS (200 ng/mL, Sigma) for 6 h, and the medium was collected as macrophage-conditioned medium (MCM) [18]. The MCM was then stored for later use after filtration.

H9C2 rat myocardial cells were obtained from the American Type Culture Collection (ATCC; Rockville, MD, USA). The cells were cultured in high-glucose DMEM supplemented with 10% (*v*/*v*) FBS, 100 U/mL penicillin, and 100 mg/mL streptomycin under a humidified atmosphere of 5% CO_2_ at 37°C. To determine the effects of *β*-OHB, the H9C2 cells were exposed to 10% MCM with or without 5 mM *β*-OHB for 24 h as described previously [18, 23]. N-acetylcysteine (NAC) (5 M, Sigma, A7250) was used as a nonspecific thiol antioxidant ROS scavenger. ITSA1 (10 *μ*M, MedChemExpress), an activator of histone deacetylases, or entinostat (250 nM, MedChemExpress), a HDAC inhibitor, was used to induce histone acetylation in H9C2 cells exposed to MCM.

### 2.3. Measurement of *β*-OHB and Acetoacetic Acid (AcAc) Levels in the Plasma and Cardiac Tissues

After 6 h of LPS injection, the blood *β*-OHB concentrations were determined from tail blood samples with a ketone meter (Precision Xtra, Abbott). The *β*-OHB and AcAc levels in frozen cardiac tissues (5–10 mg) were measured by using the Ketone Body Assay Kit (#MAK134, Sigma) according to the supplier's instructions.

### 2.4. Transthoracic Echocardiography

Transthoracic echocardiography was performed in mice using the Vevo 3100 High-Resolution In Vivo Imaging System instrument (Vevo 3100, FUJIFILM Visual Sonics, Canada) equipped with a 40-MHz MS550D scan probe. Mice were anesthetized with inhaled isoflurane (1% at 1 L/min oxygen flow), positioned supine on the animal-handling platform, with the chest area shaved and ultrasound coupling gel liberally applied to the left chest wall. Two-dimensional and M-mode echocardiographic images were recorded, and the ejection fraction (EF), fractional shortening (FS), and cardiac output (CO) were analyzed. All measurements were conducted in a blinded fashion.

### 2.5. Measurement of Creatine Kinase MB (CK-MB) and Lactate Dehydrogenase (LDH)

The blood samples were obtained via heart puncture after 6 h of LPS injection. The samples were centrifuged at 2500 rpm for 6 min at room temperature, and the plasma aliquots were collected for analysis. The concentrations of CK-MB and LDH in the plasma were measured using an automatic biochemical analyzer (BS-120; Mindray, China).

### 2.6. Histological Assessments

The mice hearts were arrested in diastole by KCl (30 mM), perfused with PBS through the heart, then fixed in 10% neutral buffered formalin for 24 h at 4°C. The hearts were embedded in paraffin and sectioned using a microtome. As for hematoxylin and eosin (H&E) staining, the left ventricle tissue sections (5-*μ*m thickness) were stained with hematoxylin for 8 min, washed with running water, and then dipped in 1% hydrochloric acid in ethanol and 1% ammonium hydroxide, successively. Then, the sections were stained with eosin for 1 min, dehydrated with gradient ethanol, permeabilized with xylene, and mounted on slides with neutral balsam. The histological changes were observed by optical microscopy (Olympus, Tokyo, Japan) with 6 random fields of vision selected from each section.

### 2.7. Transmission Electron Microscopy

The left ventricular tissues were cut into small blocks (about 1 mm^3^), fixed with 2.5% glutaraldehyde and 2% paraformaldehyde in 0.1 M cacodylate buffer for 2 h, and then fixed with 1% cold osmium tetroxide for 2 h at 4°C. After dehydrating in gradient ethanol solutions (10 min/change) and embedded in Epon 812, the tissue blocks were cut into slices with a thickness of 60 nm using the Leica Cryostat System (EM UC7/FC7). The ultrathin sections were counterstained with 3% uranyl acetate and lead citrate. Then, mitochondrial ultrastructure was acquired using a transmission electron microscope (JEM-1400PLUS, JEOL, Japan). The images were analyzed by two pathologists in a blinded fashion.

### 2.8. Assessment of Mitochondrial Oxygen Consumption Rate (OCR)

The cardiac mitochondria were isolated using differential centrifugation described previously [30]. Briefly, freshly isolated hearts were rinsed with cold sucrose-containing mitochondrial isolation buffer, chopped into pieces, and then homogenized in mitochondrial isolation buffer using the Dounce homogenizer with Teflon pestle. Mitochondria were purified by sucrose density gradient centrifugation. The oxygen consumption rates (OCR) of cardiac mitochondria or cultured cells were measured using the Seahorse XFe24 Analyzer (seahorse, Agilent Technologies), as described previously [31]. Briefly, freshly isolated mitochondria were transferred to the XFe24 microplate filled with the mitochondria assay solution (MAS: 220 mM mannitol, 70 mM sucrose, 10 mM MgCl_2_, 2 mM HEPES, 1 mM EGTA, 10 mM KH_2_PO_4_, and BSA 0.02% (*w*/*v*) at pH 7.2). The final volume was made up to 500 *μ*L per well containing 10 mM pyruvate/5 mM malate (Sigma). OCR was measured at the baseline and after an sequential injection of ADP (4 mM, Sigma), oligomycin A (2.5 *μ*g/mL, MCE), FCCP (trifluorocarbonyl cyanide phenylhydrazone, 4 *μ*М, MCE), and antimycin A (4 *μ*М, Sigma) at 37°C. For the cell OCR measurement, the H9C2 cells were maintained in a non-CO_2_ incubator at 37°C for 1 h before starting the assay. The baseline OCR measurements were obtained first, followed by sequential injection of oligomycin A (5 *μΜ* final concentration, MCE), FCCP (3 *μΜ* final concentration, MCE), and Rotone/Antimycin A (1 *μ*М/1 *μ*М final concentration, Sigma) at 37°C. The respiratory rates are reported as oxygen flux per mass, and all readings are normalized to mitochondrial protein or cell number (pmolO_2_/min/*μ*g protein or 10000 cells).

### 2.9. Measurement of ROS and Superoxide Production

As measures of oxidative stress, the superoxide dismutase (SOD) activity and malondialdehyde (MDA) levels were detected in the cardiac tissues by using commercially available kits (Beyotime, China), according to the manufacturer's recommendations. The protein carbonylation in the cardiac tissues was confirmed by measuring the protein carbonyl content using the OxyBlot Protein Oxidation Detection kit (Sigma). As for dihydroethidine (DHE) staining, the mouse cardiac tissues were embedded in the Tissue-Tek OCT compound (Sakura Finetek, Tokyo, Japan) and serially sectioned to 10-*μ*m-thickness. The cryosections were stained with the superoxide-sensitive dye DHE (10 *μ*M in 0.01% DMSO) and incubated for 30 min at 37°C in a humidified dark chamber. All sections were photographed under an inverted fluorescence microscope (IX83, Olympus, Japan). To detect mitochondrial superoxide production, H9C2 cells were incubated in a 6-well plate and stained with the Hoechst 33342 (Beyotime, #C1017) at 37°C for 20 min. After washing with PBS, H9C2 cells were incubated with the MitoSOX Red (5 *μ*M, Thermo Fisher) for 10 min at 37°C. The nuclei were visualized at Ex: 346 nm, and the level of mitochondrial superoxide was visualized at Ex: 510 nm using the Olympus IX83 microscope. As for the quantitative detection of mitochondrial superoxide, the cells were incubated on a 96-well plate and stained with 2.5-*μ*M MitoSOX Red at 37°C for 30 min, while the fluorescence intensity was quantified at 580 nm with a microplate reader (Biotek #21261610, USA). As for the MitoSOX flow cytometric analysis, H9C2 cells were incubated with 2.5 *μ*M MitoSOX Red for 30 min at 37°C, washed with PBS, and resuspended in 200 *μ*L ice-cold PBS. Flow cytometry analysis was immediately performed using the CytoFLEX flow cytometry (Beckman Coulter cytoFLEX, USA) equipped with Ex/Em: 510 nm/580 nm lasers. All of the abovementioned operations were performed without any light exposure.

#### 2.9.1. Enzyme-Linked Immunosorbent Assay (ELISA)

ELISA kits of interleukin 1 beta (IL-1*β*, Elabscience, E-EL-R0012c), interleukin-6 receptor (IL-6, Elabscience, E-EL-M2453c), and tumor necrosis factor-alpha (TNF-*α*, Elabscience, E-MSEL-M0002) were employed to detect the levels of indicated inflammatory cytokines in LPS-stimulated MCM. All ELISA kits were purchased from Elabscience Biotechnology Co., Ltd (Wuhan, China). Briefly, the standards and samples were diluted, vortexed, and centrifuged before use. Next, IL-1*β*, IL-6, and TNF-*α* were analyzed by Sandwich ELISA kits following the manufacturer's protocol. Inflammatory cytokines concentration was read immediately at 450 nm. The standard curve of each inflammatory cytokine was curve-fitted, and the unit of inflammatory cytokine was recorded in pictogram per milliliter.

### 2.10. RNA Isolation and Quantitative Real-Time PCR (qRT-PCR)

Total RNA was isolated from frozen left ventricular tissues or cultured cells with the TRIzol reagent (Thermo Fisher Scientific, Inc.). cDNA was synthesized with Omniscript reverse synthase and random hexamers according to the manufacturer's guidelines (Vazyme Biotech). All primers were purchased from the TSINGKE Biosystems. qRT-PCR was performed with the SYBR green (Bio-Rad) under a cycling protocol (40 cycles) with 10-s denaturation (95°C), 20-s annealing (60°C), and 15-s extension (72°C). The relative mRNA expression of FoxO3a and MT2 were calculated by the *^ΔΔ^*Ct method, and the results were normalized to the GAPDH levels. The primers are described as follows:

FoxO3a_F, AGGACCTGCTCACTTCGGACTC;

FoxO3a_R,CAAGGCTGCTGGACTCACTCAAG;

MT2_F, ATGGATCCCAACTGCTCCTG;

MT2_R, CGACGCCCCTTTGCAGAT;

GAPDH_F, GAAGGTGAAGGTCGGAGTC;

GAPDH_R, GAAGATGGTGATGGGATTTC.

### 2.11. Acid Extraction of Histones

The frozen heart tissues were homogenized in an ice-cold Triton extraction buffer consisting with 0.5% Triton X-100, 2 mM phenylmethylsulfonyl fluoride (PMSF), 0.02% NaN_3_, and 5 mM sodium butyrate. The lysates were mixed with vortexing for 10 min at 4°C and then centrifuged at 500 g for 10 min at 4°C. After washing twice in the Triton extraction buffer, the precipitates were then resuspended in 0.2 M HCl overnight at 4°C with mild agitation. After centrifugation at 500 g for 10 min, histones in the supernatant were harvested for further analysis. Tris-base was added to the histone fraction for neutralization before SDS-PAGE. Protein gel stained with Coomassie blue was used as the loading control (Beyotime, #P0017F, China).

### 2.12. Western Blotting

The frozen heart tissues or cultured cells were lysed with ice-cold RIPA lysis buffer supplemented with protease inhibitor cocktail (Roche), PMSF, Na_2_VO_3_, and deacetylase inhibitor cocktail (Roche). The supernatant was harvested by centrifuging at 13000 g for 15 min at 4°C. The protein was separated by SDS-PAGE and transferred onto the polyvinylidene fluoride (PVDF) membranes for immunoblotting. The PVDF membrane was blocked in 5% nonfat milk and then incubated with primary antibodies overnight at 4°C. The primary antibodies included anti-FOXO3A (10849-1-AP, Proteintech), anti-SOD2 (24127-1-AP, Proteintech), anti-GAPDH (10494-1-AP, Proteintech), anti-histone H3 (ab1791, Abcam), and anti-acetylated histone H3K9 (ab32129, Abcam) antibodies. The signal intensities were visualized by the ECL Western blotting Detection kit (#P10100, ECM) after incubation with HRP-conjugated secondary antibodies (#SA00001-1 and #SA00001-2; Proteintech) and quantified by the ImageJ software (National Institutes of Health, Bethesda, MD, USA).

### 2.13. Chromatin Immunoprecipitation (ChIP) Analysis

ChIP was performed using the High Sensitivity ChIP Assay Kit (Zoektech#A01-0325, China). According to the manufacturer's protocol, the protein-DNA complex was cross-linked with formaldehyde, sonicated, and immunoprecipitated with antibody-conjugated protein A/G agarose beads (Santa Cruz Biotechnology, Santa Cruz, CA) at 4°C overnight. The antibodies used for ChIP were normal mouse IgG (Santa Cruz Biotechnology), anti-histone H3 (ab1791, Abcam), and anti-acetylated histone H3K9 (ab32129, Abcam). The immunoprecipitated DNA was recovered with proteinase K and purified with phenol/chloroform precipitation. The precipitated DNA was analyzed with SYBR green qPCR. As for the H3K9 promoter binding assay, the primer sets used as follows:

FOXO3A-0.4 K-F,GGTTTTCTTGCAGTCCGAGAG;

FOXO3A-0.4 K-R,GACCCGCTAAGAAGATCTGAGG;

FOXO3A-0.6 K-F,GGGTCGGTCCTCTCTCGTT;

FOXO3A-0.6 K-R,GCAACTCCCGTCTTTTCCTC;

FOXO3A-1 k-F,TCCCCAGGTCTATCCGTTCT;

FOXO3A-1 K-R,GGGCTCTTGCTCTCTCCTCT;

FOXO3A-1.2 K-F,AACTCTCTCTTCGCGCTTCC;

FOXO3A-1.2 K-R,TTGGGCTCTTGCTCTCTCCT;

MT2-0.1 K-F,AGAGCAGGACGGACTTTTG;

MT2-0.1 K-R,CGAGCCATTATCTCAAGGACT;

MT2-0.2 K-F,GGGTACTACTGGCTGCTCCT;

MT2-0.2 K-R,CAAAGAACTCGGGTGCAAG.

#### 2.13.1. Statistical Analysis

GraphPad Prism v.9.0.1 (GraphPad, La Jolla, CA) was employed for statistical analysis. The Shapiro-Wilk normality test was performed to determine the data distribution. All data were normally distributed and presented as the mean ± standard deviation (SD). Paired or unpaired two-tailed Student's *t*-tests were selected to compare two groups. One-way analysis of variance (ANOVA), followed by Tukey's multiple comparisons test, was performed to determine the differences among > 2 groups. *P* < 0.05 was considered to be statistically significant.

## 3. Results

### 3.1. Ketone Bodies Protected the Hearts against LPS-Induced Acute Myocardial Injury and Cardiac Dysfunction

**Figure 1:**
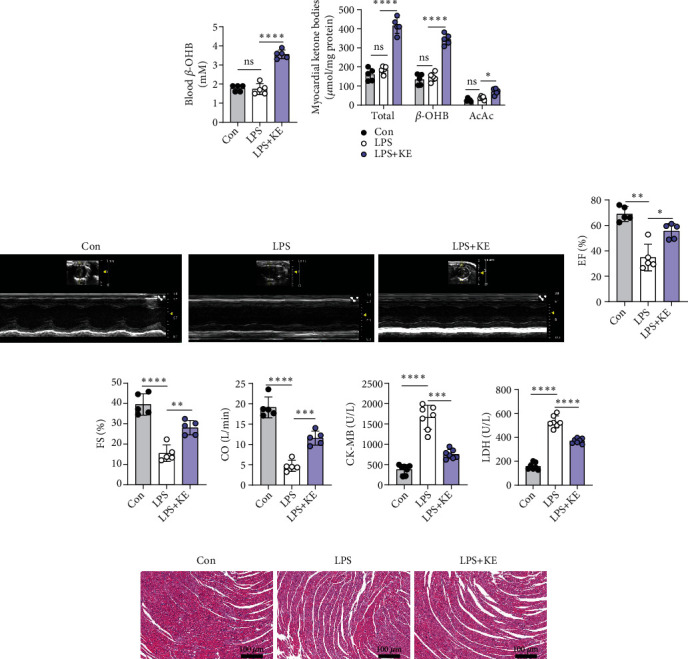
Ketone bodies protected the hearts against LPS-induced acute myocardial injury and cardiac dysfunction. (a). Blood *β*-OHB levels in Con, LPS, and LPS + KE mice (*n* = 5 per group). (b). The levels of ketone bodies (*β*-OHB; AcAc) in the Con, LPS, and LPS + KE hearts (*n* = 5 per group). (c). Representative echocardiography images of the Con, LPS, and LPS + KE hearts (*n* = 5 per group). (d–f) Cardiac function was indicated by ejection fractions (EF) (d), shortening fraction (FS) (e), and cardiac output (CO) (f) (*n* = 5 per group). (g and h) The levels of CK-MB (g) and LDH (h) in the plasma samples (*n* = 7 per group). (i) Representative images of hematoxylin and eosin- (H&E-) stained left ventricle tissue sections (*n* = 5 per group). Scale bar = 100 *μ*m. Con, Control; LPS, lipopolysaccharide; and KE, ketone ester. Data are presented as the mean ± SD. Statistical comparisons were conducted by one-way ANOVA, followed by Tukey's multiple comparisons test (a, b, d, e, f, g, and h). The exact *P* values were reported for the indicated comparisons, and *P* < 0.05 was considered statistically significant. ^∗^*P* < 0.05, ^∗∗^*P* < 0.01, ^∗∗∗^*P* < 0.001, and ^∗∗∗∗^*P* < 0.0001 for the indicated comparisons. ns: no significant difference.

Male C57BL/6 J mice were exposed to LPS (20 mg/kg, i.p.) to induce septic cardiomyopathy, as described previously [5, 8]. In the KE pretreatment group, an almost two-fold increase was detected in the levels of circulating ketone bodies, especially that of *β*-OHB (Figure 1(a)). Remarkably, the levels of total ketone bodies and their two primary forms, namely, *β*-OHB and AcAc, increased in the heart tissues (Figure 1(b)), which indicated that KE gavage significantly enhanced the levels of ketone bodies both in the bloodstream and in the heart tissues (Figures 1(a) and 1(b)).

Six hours after LPS injection, echocardiography revealed that the LPS-treated mice demonstrated a sharp decrease in LVEF, FS, and CO (Figures 1(c)–1(f)), which established that LPS-induced acute septic cardiomyopathy. Consistently, the plasma levels of cardiac damage markers, CK-MB and LDH (Figures 1(g) and 1(h)) were substantially higher in the LPS-treated mice compared with the control group. These results suggested that LPS-induced myocardial injury and reduced cardiac function. Compared with the LPS group, the mice in the LPS + KE group appeared to have increased LVEF, FS, and CO (Figures 1(c)–1(f)) and decreased levels of cardiac damage markers (Figures 1(g) and 1(h)), which implied that the ketone bodies mitigated heart injury and preserved heart contractile function (Figures 1(c)–1(h)).

Furthermore, H&E staining indicated that the myocardial cells were sparse and disordered, with interstitial edema and abundant inflammatory cell infiltration in the LPS group compared with the control group (Figure 1(i)). Interestingly, KE treatment ameliorated the structural disorderliness in the heart of LPS-treated mice, which meant that the cardiomyocytes were more organized and have fewer inflammatory cell infiltrates and granulocytic aggregates (Figure 1(i)). These results suggest that the ketone bodies prevented LPS-induced septic myocardial injury and cardiac dysfunction.

### 3.2. Ketone Bodies Mitigated Cardiac Mitochondrial Dysfunction and Oxidative Stress in Septic Cardiomyopathy

**Figure 2:**
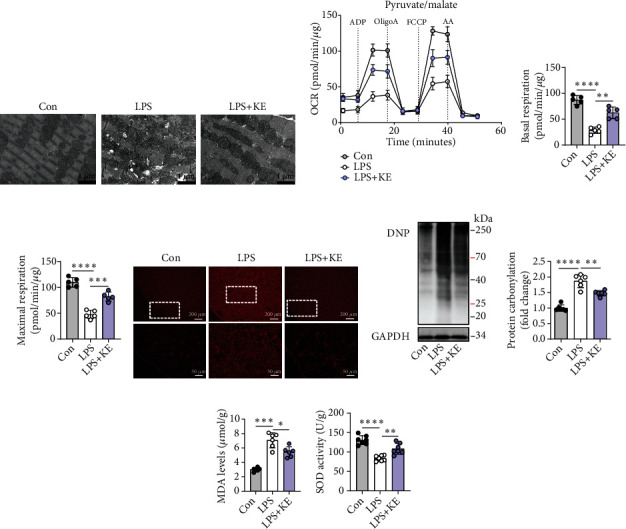
Ketone bodies mitigated cardiac mitochondrial dysfunction and oxidative stress in septic cardiomyopathy. (a) Representative transmission electron microscopy images of the Con, LPS, and LPS + KE heart sections (*n* = 5 per group). Scale bar = 1 *μ*m. (b–d) The oxygen consumption rate (OCR) curve in the presence of pyruvate and malate of the isolated mitochondria from the mouse hearts (b). The ADP-stimulated OCR (c) and FCCP-stimulated OCR (d) (*n* = 5 per group). (e) Representative images of dihydroethidium (DHE) staining in Con, LPS, and LPS + KE hearts (*n* = 50 fields, from 3 hearts per group). Scale bar = 200 *μ*m or 50 *μ*m. (f and g) Western blotting of 2,4-dinitrophenylhydrazone (DNP) in the heart tissues from Con, LPS, and LPS + KE mice (f) (*n* = 5 per group). The protein levels of DNP were normalized to the GAPDH levels, and the data were expressed as the fold change relative to the control (g) (*n* =5 per group). (h) Malondialdehyde (MDA) levels in the heart tissues (*n* = 5 per group). (i) Superoxide dismutase (SOD) activity in the heart tissues (*n* = 5 per group). Con: Control; LPS: lipopolysaccharide; KE: ketone ester; ADP: adenosine diphosphate; Oligo: oligomycin A; FCCP: trifluorocarbonyl cyanide phenylhydrazone; and AA: antimycin A. Data are presented as the mean ± SD. Statistical comparisons were conducted using one-way ANOVA, followed by Tukey's multiple comparisons test (c, d, g, h, and i). The exact *P* values were reported for the indicated comparisons, with *P* < 0.05 considered statistical significance. ^∗^*P* < 0.05, ^∗∗^*P* < 0.01, ^∗∗∗^*P* < 0.001, and ^∗∗∗∗^*P* < 0.0001 for the indicated comparisons.

Mitochondrial abnormalities are closely related to cardiac dysfunction [15, 32]. To visualize the ultrastructural alterations of the mitochondrion in the hearts of LPS-treated mice, we conducted transmission electron microscopic analyses. The hearts of LPS-treated mice showed notable cytoarchitectural destruction, especially disrupted mitochondria, as shown with mitochondrial swelling along with the formation of internal vesicles, disorganized cristae, and lowered matrix density (Figure 2(a)). Intriguingly, pretreatment with ketone ester preserved the heart mitochondrial ultrastructure to some extent (Figure 2(a)). It is well known that the disruption of mitochondrial morphological structure and functional dysregulation often occur simultaneously. Correspondingly, seahorse analysis confirmed that LPS induced an apparent decrease in pyruvate/malate-supported mitochondrial respiratory function (Figures 2(b)–2(d)), while ketone bodies improved both the ADP-stimulated basal OCR and FCCP-stimulated maximal OCR (Figures 2(b)–2(d)).

The mitochondrion is the primary source of intracellular ROS and is extremely susceptible to oxidative stress. Therefore, we investigated whether the benefit offered by the ketone bodies was mediated by suppressing oxidative stress. The fluorescence intensity of DHE-stained cardiac tissue increased in the LPS group, which suggested a high level of ROS production (Figure 2(e)). Moreover, excessive protein carbonylation, an irreversible oxidative protein modification (Figures 2(f) and 2(g)), increased level of MDA, and decreased SOD activity were observed in the hearts of LPS-treated mice (Figures 2(h) and 2(i)). Notably, the LPS + KE hearts showed reduced fluorescence intensity of DHE, accompanied by mitigated protein carbonylation, lowered MDA level, and enhanced SOD activity (Figures 2(e)–2(i)). These results suggest that while LPS-induced septic heart injury was associated with oxidative stress damage, ketone bodies mitigated cardiac oxidative stress, and improved mitochondrial function in septic cardiomyopathy.

### 3.3. *β*-OHB Alleviated Oxidative Stress and Enhanced Aerobic Respiration in H9C2 Cells Exposed to MCM

**Figure 3:**
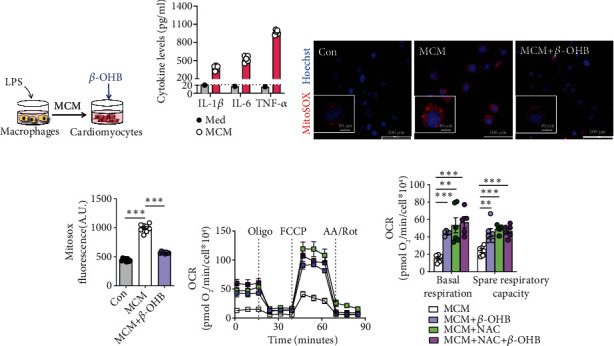
*β*-OHB alleviated oxidative stress and enhanced aerobic respiration in H9C2 cells exposed to MCM. (a) Schematic representation of the experimental protocol. (b) The levels of inflammatory cytokines in LPS-stimulated macrophage conditioned medium (MCM) (*n* = 5) and standard cell culture medium (DMEM, Med) with 10% fetal calf serum. (c). Representative fluorescent images of H9C2 cells stained with MitoSOX red after the indicated treatments. Scale bar = 100 *μ*m. The nuclei were visualized with Hoechst staining. Scale bar = 10 *μ*m. (d). The fluorescence intensity of MitoSOX-stained H9C2 cells after the indicated treatments (*n* = 6 per group). (e and f) The oxygen consumption rate (OCR) curve in the presence of pyruvate and L-glutamine in H9C2 cells after the indicated treatments (e) and the basal respiration and spare respiratory capacity (OCR_FCCP_–OCR_basal_) (f) (*n* = 5–6 per group). Oligo: Oligomycin A; A A./Rot: antimycin A and rotenone. Data are presented as the mean ± SD. Statistical comparisons were conducted by one-way ANOVA, followed by Tukey's multiple comparisons test (b, d, and f). The exact *P* values are reported for the indicated comparisons, and *P* < 0.05 indicates statistical significance. ^∗∗^*P* < 0.01 and ^∗∗∗^*P* < 0.001 for the indicated comparisons.

An excessive inflammatory response has been acknowledged as the hallmark of septic cardiomyopathy [3, 9]. In sepsis, inflammatory infiltration caused by macrophages secreting several cytokines is the leading cause of myocardial injury [3, 4]. To verify the effect of ketone bodies on myocardial mitochondrial oxidative stress, H9C2 cells were incubated with LPS-stimulated MCM to mimic the pathological process of septic cardiomyopathy and were treated with *β*-OHB for 24 h (Figure 3(a)). ELISA proved that the levels of inflammatory cytokines, including IL-1*β*, IL-6, and TNF-*α*, were drastically elevated in the MCM-cultured cells (Figure 3(b)).

Moreover, the intensity of MitoSOX fluorescence, an indicator of mitochondrial superoxide, was remarkably enhanced in the MCM-cultured H9C2 cells. Meanwhile, *β*-OHB treatment mitigated ROS production (Figures 3(c) and 3(d)). Additionally, seahorse analysis showed that the mitochondrial respiratory function of H9C2 cells was also improved by *β*-OHB, indicated with both basal respiration and spare respiratory capacity (Figures 3(e) and 3(f)). Intriguingly, the additive effect of *β*-OHB was not observed, and the nonspecific ROS scavenger N-acetylcysteine (NAC) in the H9C2 cells exposed to MCM suggested that *β*-OHB exhibited antioxidant effects similar to those of NAC on cellular aerobic respiration (Figures 3(e) and 3(f)). Together, these data imply the protective effect of *β*-OHB as an antioxidant on myocardial oxidative stress and mitochondrial function.

### 3.4. *β*-OHB Alleviated Oxidative Stress and Improved Mitochondrial Function via HDAC Inhibition in MCM-Cultured H9C2 Cells

**Figure 4:**
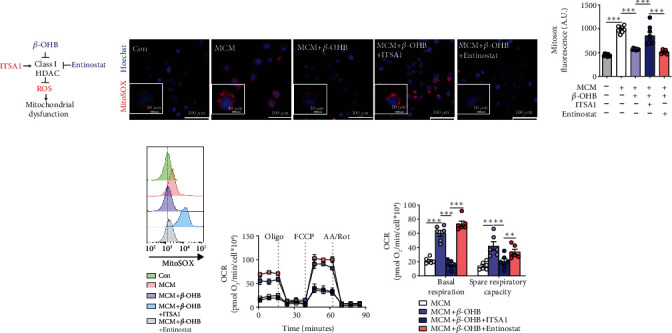
*β*-OHB alleviated oxidative stress and improved mitochondrial function via HDAC inhibition in MCM-treated H9C2 cells. (a) ITSA1 (HDAC activator) and entinostat (HDAC inhibitor) were used in this study. (b) Representative fluorescent images of H9C2 cells stained with MitoSOX red to assess the mitochondrial ROS generation after the indicated treatments (*n* = 8 per group). Scale bar = 100 *μ*m. The nuclei were visualized with Hoechst staining. Scale bar = 10 *μ*m. (c). The fluorescence intensity of MitoSOX-stained H9C2 cells after the indicated treatments (*n* = 8 per group). (d). Representative MitoSOX flow cytometry analysis in H9C2 cells after the indicated treatments (*n* = 6 per group). (e and f) The oxygen consumption rate (OCR) curve in the presence of pyruvate and L-glutamine in H9C2 cells after the indicated treatments (e), and the basal respiration and spare respiratory capacity (OCR_FCCP_−OCR_basal_) (f) (*n* = 4 per group). Oligo: Oligomycin A; A.A./Rot: antimycin A and rotenone. Data are presented as the mean ± SD. Statistical comparisons were conducted by one-way ANOVA, followed by Tukey's multiple comparisons test (c and f). The exact *P* values are reported for the indicated comparisons, and *P* < 0.05 indicates statistical significance. ^∗∗^*P* < 0.01 and ^∗∗∗^*P* < 0.001 for the indicated comparisons.

As an endogenous and specific inhibitor of HDAC, *β*-OHB has been reported to confer significant resistance against oxidative stress [23] (Figure 4(a)). To identify whether the protective effect of *β*-OHB on mitochondrial function and oxidative stress was mediated by histone acetylation in septic cardiomyopathy, ITSA1, an activator of histone deacetylase, or entinostat, an inhibitor of HDAC was used to regulate histone acetylation in MCM-cultured H9C2 cells. It is worth noting that the suppression of mitochondrial superoxide by *β*-OHB could be abolished by ITSA1 or mimicked by entinostat (Figures 4(b)–4(d)), which indicated that *β*-OHB inhibited mitochondrial ROS production in response to histone acetylation status (Figures 4(b)–4(d)).

As expected, seahorse analysis showed that the improvement of cellular aerobic respiration in MCM-cultured H9C2 cells by *β*-OHB was similar to that of entinostat but opposite to that of ITSA1 (Figures 4(e) and 4(f)), thereby signifying that *β*-OHB improved mitochondrial function. Collectively, these results demonstrated that *β*-OHB protected the mitochondria against oxidative stress damage and improved mitochondrial function via HDAC inhibition in MCM-cultured H9C2 cells.

### 3.5. Ketone Bodies Enhanced FoxO3a And MT2 Expressions by Promoting Histone H3 Lysine 9 Acetylation

**Figure 5:**
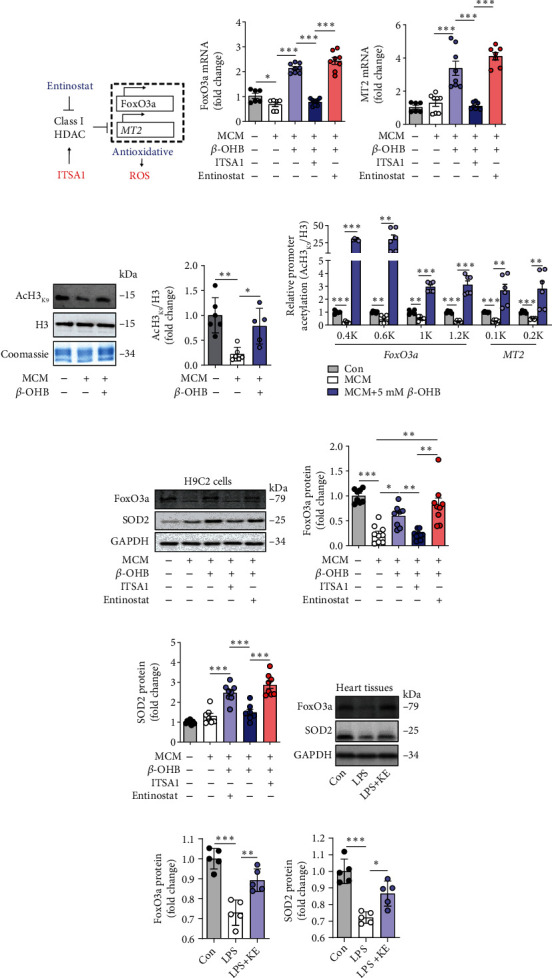
The ketone bodies enhanced FoxO3a and MT2 expression by promoting histone H3 lysine 9 acetylation. (a) HDAC exerts oxidative stress resistance through the FoxO3a/MT2 pathway. FoxO3a: Forkhead box O3; MT2: Metallothionein 2A. (b and c) qRT-PCR analysis of *FoxOa3* (b) and *MT2* (c) in H9C2 cells after the indicated treatments (*n* = 6–8 per group). (d and e) Western blotting of the acetylation of H3K9 in purified histones from H9C2 cells after the indicated treatments (d). The quantification of the band intensity (e). Acetylation is normalized to the total histone content H3 and reported to be relative to the MCM-exposed cells. Coomassie blue staining was used as the gel loading control (*n* = 5–6 per group). (f) Chromatin from H9C2 cells after the indicated treatments were immunoprecipitated with anti-histone H3 or anti-AcH3K9, and the purified DNA was analyzed with primer pairs specific for the *FoxO3a* or *MT2* promoters. The results are shown as the ratios of AcH3K9 to total histone H3 (*n* = 5–6 per group). (g–i) Western blotting of FoxO3a and SOD2 in H9C2 cells after the indicated treatments (g). Quantification of the band intensity (h and i). The protein levels of FoxO3a and SOD2 were normalized to those of GAPDH, and the data are expressed as fold changes relative to the control value (*n* = 7–9 per group). (j–l) Western blotting of FoxO3a and SOD2 in the heart tissues from control, LPS, and LPS + KE mice (j). Quantification of the band intensity (k and l). The protein levels of FoxO3a and SOD2 were normalized to those of the GAPDH, and the data are expressed as the fold change relative to the control value (*n* = 5 per group). Data are presented as the mean ± SD. Statistical comparisons were conducted using one-way ANOVA, followed by Tukey's multiple comparisons test (b, c, d, e, f, h, i, k, and l). The exact *P* values are reported for the indicated comparisons, and *P* < 0.05 indicates statistical significance. ^∗^*P* < 0.05, ^∗∗^*P* < 0.01, and ^∗∗∗^*P* < 0.001 for the indicated comparisons.

HDAC and histone acetyltransferase (HAT) are responsible for reversible changes in the histone acetylation status. Inhibition of HDAC by *β*-OHB was correlated with global changes in transcription, including genes encoding of the oxidative stress resistance factors, *FoxO3a*, and *MT2* [23, 33, 34] (Figure 5(a)). The mRNA levels of *FoxO3a* and *MT2* were estimated in the H9C2 cells. Remarkably, the expressions of *FoxO3a* and *MT2* mRNA were significantly decreased in MCM-cultured H9C2 cells, while both were elevated after treatment with 5 mM *β*-OHB (Figures 5(b) and 5(c)). To comprehend the biological impact of *β*-OHB on antioxidant genes, *β*-OHB was used, in combination with the HDAC activator ITSA1 or the inhibitor entinostat, to detect the expressions of *FoxO3a* and *MT2*. ITSA1 blocked the upregulated expressions of *FoxO3a* and *MT2* induced by *β*-OHB (Figures 5(b) and 5(c)). In contrast, entinostat did not alter the effect of *β*-OHB on the expressions of these genes (Figure 5(b) and 5(C)), which confirmed that *β*-OHB offered resistance against oxidative stress via HDAC inhibition.

Accumulating evidence suggests that histone acetylation promotes the binding of *FoxO3a* and *MT2* to their promoters and enhances their expression [23, 33]. AcH3_K9_ has been identified to play a crucial role in activating gene expression [35, 36]. Correspondingly, Western blot analysis of AcH3_k9_ in the purified histone showed that *β*-OHB triggered histone acetylation in MCM-cultured H9C2 cells (Figures 5(d) and 5(e)). Furthermore, ChIP analysis of the *Foxo3a* and *MT2* promoters with several distinct primer pairs revealed increased AcH3_K9_ binding to these gene promoters under high *β*-OHB exposure (Figure 5(f)), which confirmed the role of *β*-OHB in epigenetics.

Subsequently, the protein expressions of FoxO3a and the downstream target SOD2, which contributes to antioxidative protection, were upregulated by *β*-OHB in MCM-cultured H9C2 cells, having the same effect as that of an AcH3_K9_-induced active promoter (Figures 5(g)–5(i)). As expected, ITSA1 abrogated the promoting effects of *β*-OHB on protein expression, whereas entinostat exerted the same effect as *β*-OHB (Figures 5(g)–5(i)). Furthermore, elevated expressions of FoxO3A and SOD2 were found in septic hearts, thereby, supporting the in vitro findings (Figures 5(j)–5(l)).

Collectively, these data suggested that *β*-OHB acts as an HDAC inhibitor, which enhances histone acetylation, to activate the antioxidant FoxO3a/MT2 pathway, thereby preventing ROS production and offering mitochondrial protection in septic cardiomyopathy.

## 4. Discussion

**Figure 6:**
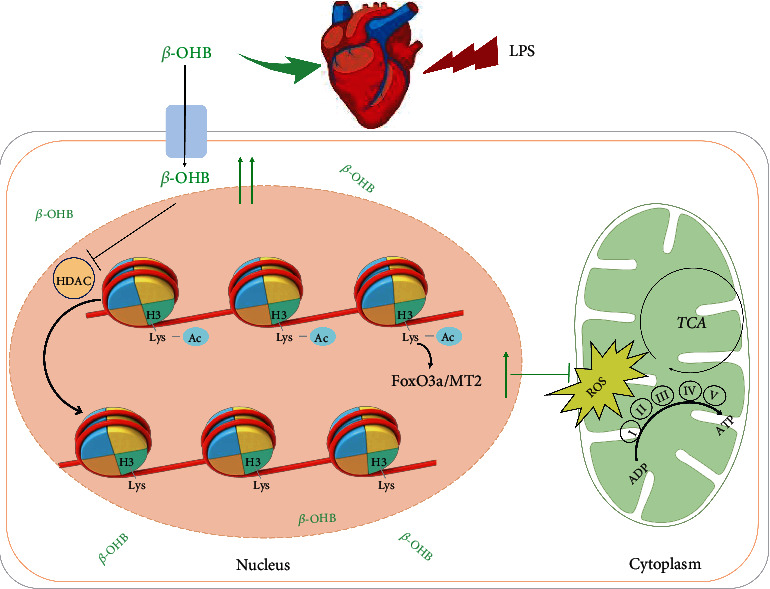
Schematic illustration of the working model for this study. *β*-OHB is an HDAC inhibitor that enhances histone acetylation to activate the antioxidant FoxO3a/MT2 pathway for preventing ROS production from protecting the mitochondria in septic cardiomyopathy.

In this study, the ketone body *β*-OHB was shown to prevent LPS-induced myocardial injury and cardiac dysfunction. Mechanistically, as an HDAC inhibitor, *β*-OHB activated the antioxidant FoxO3a/MT2 pathway. This response enhanced redox homeostasis and improved mitochondrial function (Figure 6). These findings, therefore, suggest that *β*-OHB is an effective agent for myocardial protection in sepsis.

During sepsis, a sharp decrease in the contractile function of the heart and severe myocardial damage were observed. Fortunately, the disruption of structure and the disorder of function in the septic hearts could be prevented by KE preconditioning. In the normal physiological conditions, the cardiac contractive function is highly energy consuming and depends much on mitochondrial OXPHOS for ATP generation [37, 38]. Well-organized cristae and large mitochondrial matrices are indispensable for supercomplex formation and are closely associated with electron transfer efficiency [39, 40]. In line with previous studies [9, 15], disrupted mitochondrial ultrastructure and depressed mitochondrial OXPHOS were observed in the hearts of LPS-treated mice. The preconditioning of KE contributed to high levels of *β*-OHB, which ameliorated the damage to the mitochondrial ultrastructure of the myocardium. Furthermore, *β*-OHB improved pyruvate/malate-supported mitochondrial respiration, which rescued ATP production in the septic hearts. In addition, *β*-OHB reduced global carbonylation proteins and lipid oxidation levels and increased SOD2 activity, indicating that *β*-OHB prevents ROS overproduction for mitochondrial protection. Therefore, this study provides direct evidence to support that *β*-OHB exerts a protective effect in septic cardiomyopathy and that the primary regulatory machinery fine-tunes mitochondrial performance and ameliorates oxidative stress.

Ketone bodies are essential energy sources for life activities, and *β*-OHB accounts for approximately 70% of the total circulating ketones [21]. The level of *β*-OHB in the bloodstream increases to 1–2 mM or even higher (6–8 mM) during prolonged fasting, while the concentration could be increased to >25 mM in diabetic ketoacidosis [18, 21]. Intravenously administered *β*-OHB could rapidly increase the *β*-OHB concentrations to >2 mmol/L without exerting any deleterious side effects, ascertaining the potential benefit of *β*-OHB in specific disease states, such as stroke or traumatic brain injury [41]. In chronic heart failure, myocardial ischemia and diabetic cardiomyopathy, as an energy source, *β*-OHB can exert beneficial effects to meet myocardial oxygen demand [20, 42]. *β*-OHB restored doxorubicin-induced left ventricular dysfunction and prevented cardiac remodeling through sustaining mitochondrial membrane potential integrity [43]. However, most studies have focused on the effects of *β*-OHB in case of long-term exposure [44]. In contrast, septic cardiomyopathy is an acute cardiac injury characterized by a sudden decline in LVEF in 20% of patients in the first 6 h of septic shock [3]. Overtime, the release of inflammatory factors attacks the myocardial mitochondria and results in ROS outbreak [45]. Therefore, in this study, the biochemical advantages of acute ketosis in septic cardiomyopathy were explored using short-term KE supplements. Interestingly, the ingestion of KE by LPS-challenged mice for 3 days and the exposure of MCM-cultured H9C2 cells to high concentrations of *β*-OHB resulted in reduced ROS production. Our results demonstrated that *β*-OHB is an effective and practical treatment for improving myocardial mitochondrial performance in septic cardiomyopathy.

In addition to serving as alternative biofuel for extrahepatic tissues, for example, the heart, brain, and skeletal muscle, ketone bodies have also been shown to play pivotal roles in signal transduction, protein post-translational modification, and inflammation regulation [23, 46]. In recent years, *β*-OHB has been shown to be a novel HDAC inhibitor owing to its structural similarity with butyrate (a traditional HDAC inhibitor) [23]. AcH3_K9_ is crucial and linked to the active promoter [36]. Hence, the histone acetylation levels of AcH3_K9_ were measured, and *β*-OHB was found to trigger histone acetylation, which is consistent with the results of a previous experiment on human embryonic kidney cells (HEK293) [23]. The role of AcH3_K9_-activated transcription factor FoxO3a has been associated with antioxidant stress in various organisms [47]. In our study, ChIP analysis of the FoxO3a and MT2 promoters revealed increased AcH3_K9_ due to high *β*-OHB exposure. The *FoxO3a* and *MT2* genes, which encode antioxidant proteins, for example, SOD and catalase dismutase, are activated when histone acetylation increases [14, 48]. At the protein level, *β*-OHB was noted to enhance the expressions of FoxO3a and SOD2 in septic hearts. Manabu Nagao et al. reported that the accumulation of *β*-OHB in the failing hearts occurs as a compensatory response to oxidative stress [49]. Both these studies reported an antioxidative effect of *β*-OHB in the stressed hearts; however, the key findings of the two studies were different. The Nagao paper suggested that the endogenous accumulated *β*-OHB in response to pressure overload or H_2_O_2_ exposure could protect cardiomyocytes, while our study demostrated the therapeutic effects of *β*-OHB supplementation on LPS-induced septic cardiomyopathy. Importantly, we provided new mechanistic insights by which *β*-OHB alleviated oxidative stress and improved the mitochondrial function in septic hearts (inhibition of HDAC by *β*-OHB to promote H3K9 acetylation). Above all, our findings demonstrated that *β*-OHB, an endogenous HDAC inhibitor, mediates antioxidant gene expression, and integrates redox homeostasis with epigenetic alterations. This finding adds a new mechanism to the reported function of *β*-OHB as a signaling molecule in septic cardiomyopathy.

Our study has several strengths. We pioneered the use of ketone bodies as a pharmacologic approach to prevent systolic myocardial dysfunction and rescue poor heart pumping ability in septic cardiomyopathy, thereby providing a basis for clinical therapy. Subsequently, state-of-the-art techniques, such as seahorse mitochondrial analysis and ChIP analysis, were exploited to uncover the role of *β*-OHB in signal translation and protein modification. However, we acknowledge that there are several limitations in the present study. First, the critical impacts of *β*-OHB on antioxidants in septic cardiomyopathy were not tested in vivo, such as *β*-OHB dehydrogenase (BDH1)-knockout mice, to verify the outcomes. Second, the effects of prolonged ketone body exposure and the duration of these effects after withdrawal were not investigated in this study. Third, *β*-OHB-initiated cardiac effects exhibit sex differences [5, 26]. Hence, caution should be exercised while extrapolating our findings to females and other mouse strains.

## 5. Conclusions

Our study has demonstrated that ketone bodies, especially *β*-OHB, activate antioxidant pathways by acting as HDAC inhibitors and improve mitochondrial respiratory function, thus offering functional cardiac protection in septic cardiomyopathy. These promising findings suggest the possibility of applying ketone bodies for the clinical treatment of septic heart injury.

## Data Availability

The data that support the findings of this study are available from the corresponding author on reasonable request.

## Supplementary Materials

Supplementary Materials Supplementary file.pdf: the unedited gels for this study.
