# Supplementary material for: Ketone Body β-Hydroxybutyrate Prevents Myocardial Oxidative Stress in Septic Cardiomyopathy
Source: Oxid Med Cell Longev. 2022 Mar 18;2022:2513837. doi: 10.1155/2022/2513837 (PMC8956399; doi:10.1155/2022/2513837)
Supplement: Supplementary Materials — Supplementary file.pdf: the unedited gels for this study. [file 2513837.f1.pdf]

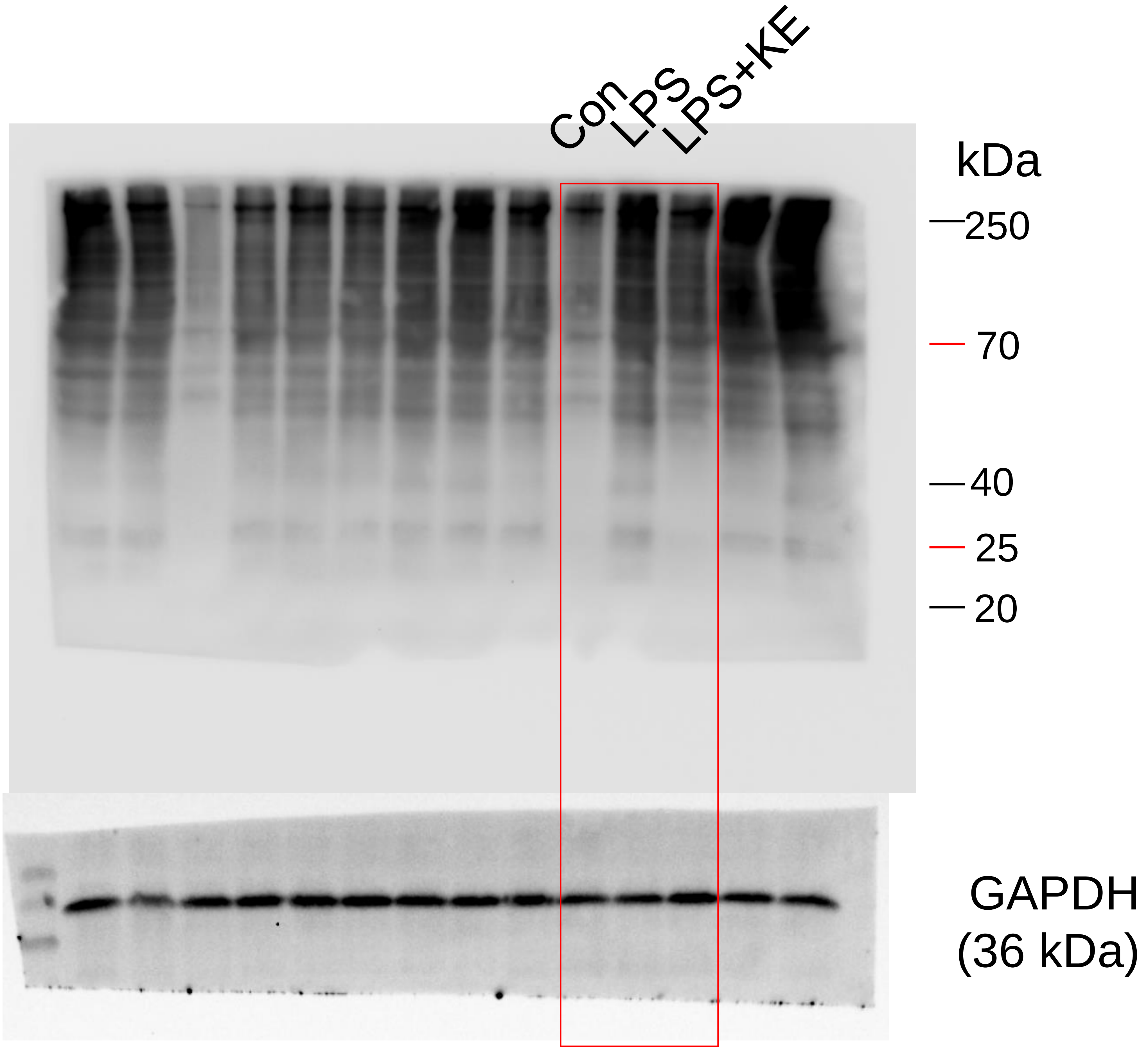

Full unedited gel for Figure 2F
Acquired with ChemiDoc (BioRad)

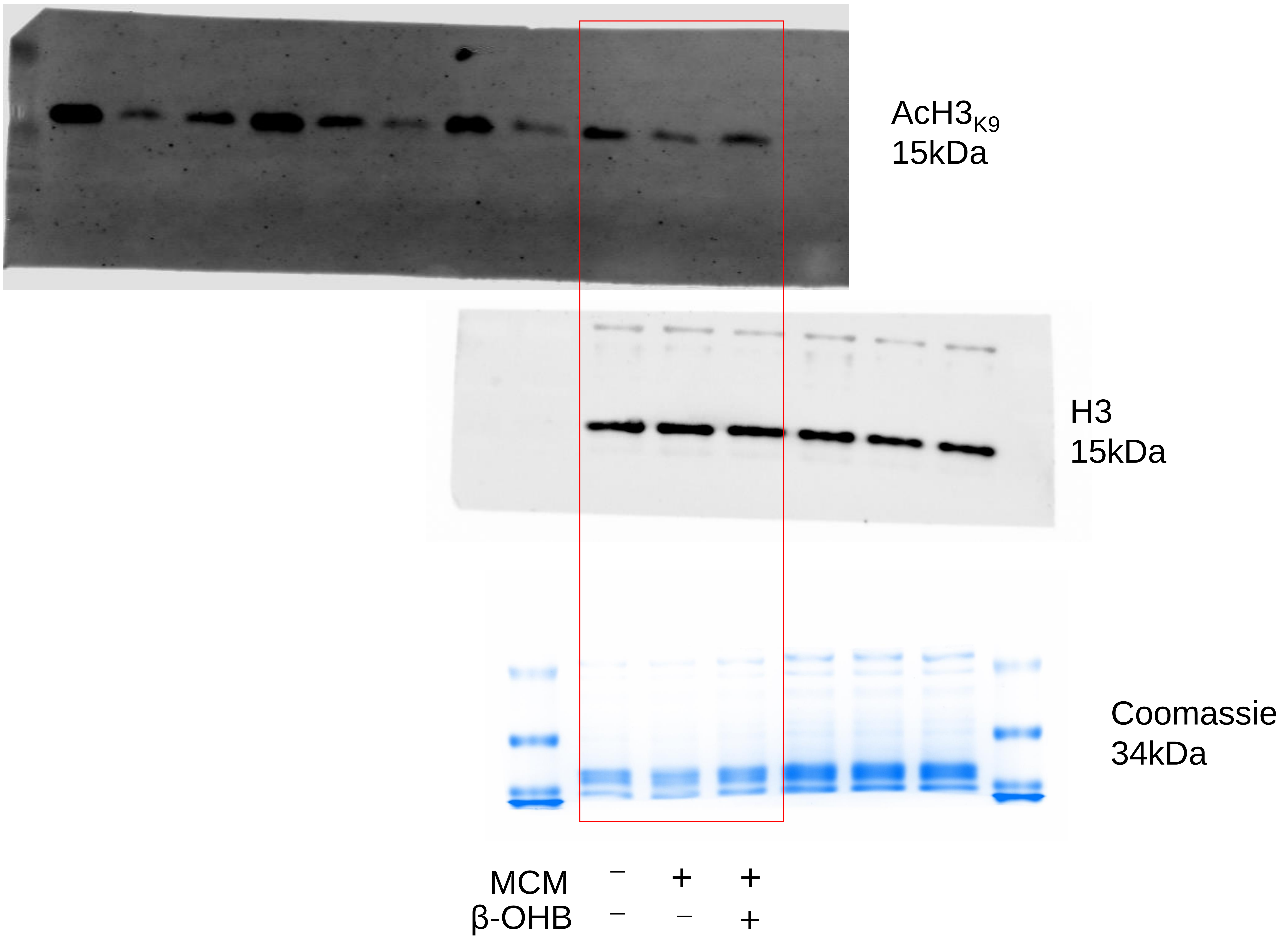

Full unedited gel for Figure 5D
Acquired with ChemiDoc (BioRad)

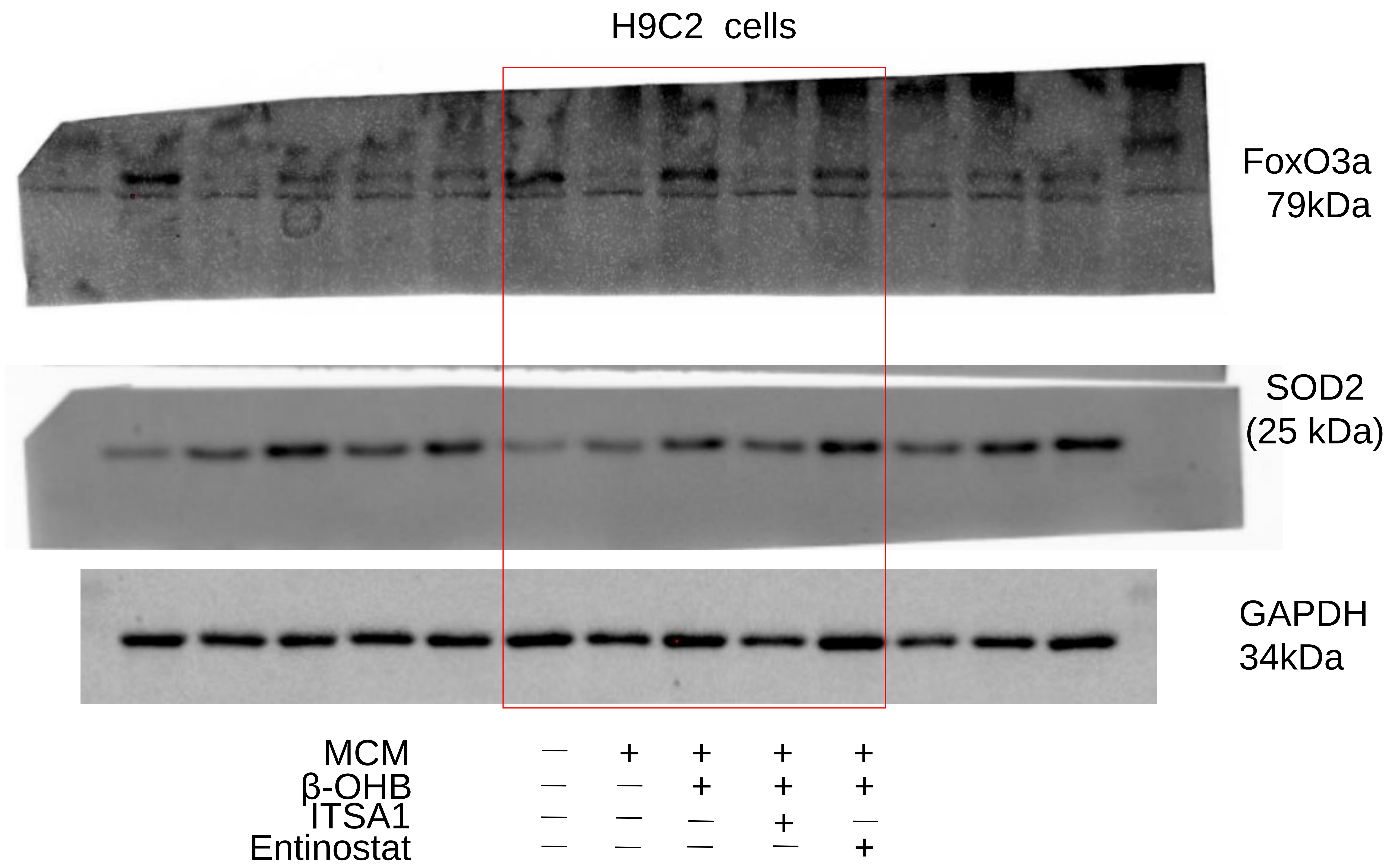

Full unedited gel for Figure 5G
Acquired with ChemiDoc (BioRad)

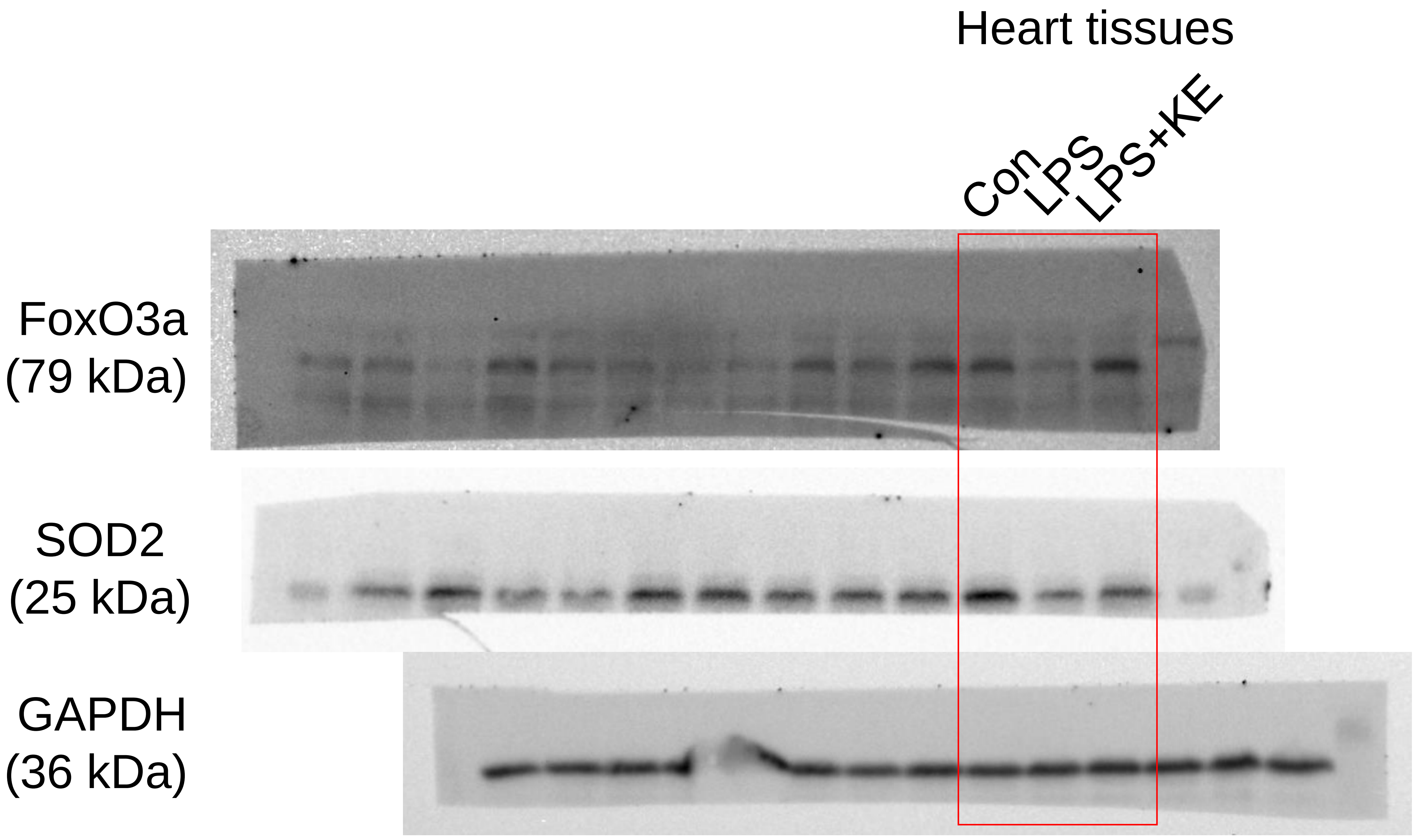

Full unedited gel for Figure 5J
Acquired with ChemiDoc (BioRad)
